# The CHEK2 1100delC allelic variant is not present in familial and sporadic breast cancer cases from Moroccan population

**DOI:** 10.1186/s40064-014-0778-5

**Published:** 2015-02-01

**Authors:** Chaymaa Marouf, Omar Hajji, Brehima Diakité, Amal Tazzite, Hassan Jouhadi, Abdellatif Benider, Sellama Nadifi

**Affiliations:** Laboratory of Genetics and Molecular Pathology–Medical School of Casablanca, 19 rue Tarik Ibn Ziad, P.C 9154 Casablanca, Morocco; University Hassan II Ain Chock, Center Of Doctoral Sciences “In Health Sciences”, Casablanca, Morocco; Department of Oncology, Littoral Clinic, Casablanca, Morocco; Mohammed VI Center for Cancer Treatment, Ibn Rochd University Hospital, Casablanca, Morocco

## Abstract

**Purpose:**

The cell-cycle checkpoint kinase 2 (CHEK2) is an important signal transducer of cellular responses to DNA damage, whose defects has been associated with increased risk for breast cancer. The CHEK2 1100delC mutation has been reported to confer a twofold increased risk of breast cancer among carriers. The frequency of the mutation varies among populations. The highest frequency has been described in Northern and Eastern European countries. However, the 1100delC mutation has been investigated in different case-control studies and none in Moroccan population. The aim of this study was to evaluate the prevalence of this variant and determine its contribution to the development of breast cancer in sporadic cases and also in members of breast cancer families who tested negative or positive for a deleterious mutation in BRCA1/BRCA2.

**Methods:**

In this case-control study we performed the CHEK2 1100delC mutation analysis by ASO-PCR in 134 breast cancer patients and 114 unaffected control individuals. Most of these families had several cases of breast cancer or ovarian cancer (or both).

**Results:**

No CHEK2 1100delC mutations were detected in any of 134 individuals, including 59 women diagnosed with breast cancer at an early age (<40 years), 10 women with bilateral breast cancer, and 6 women with ovarian cancer.

**Conclusion:**

Our preliminary genetic analysis are consistent with the reported very low frequency of CHEK2 1100delC mutation in North American populations (compared with Northern Europe), rendering CHEK2 1100delC such as an unlikely to be major breast cancer susceptibility genes.

## Introduction

Cell-cycle checkpoint kinase 2 (CHEK2 [MIM 604373]) is a tumor suppressor gene widely researched as a strong candidate gene for breast cancer susceptibility (Iniesta et al. 2010; Vahteristo et al. 2002; CHEK2 Breast Cancer Case–control Consortium CHEK2*1100delC and susceptibility to breast cancer 2004; Mateus Pereira et al. 2004; Offit et al. 2003; Zhang et al. 2008; Rashid et al. 2005; Kleibl et al. 2005; Cybulski et al. 2007; Friedrichsen et al. 2004; Bernstein et al. 2006; Sodha et al. 2002; McInerney et al. 2009; Dufault et al. 2004; Einarsdottir et al. 2006; Weischer et al. 2007; Baeyens et al. 2005; Fletcher et al. 2009). The CHEK2 gene has been shown to play a major role in deoxyribonucleic acid (DNA) repair, apoptosis and cell cycle regulation. Indeed, in response to DNA damage, the ATM/CHEK2/p53 pathway is activated. The ATM (Ataxia Telangiectasia Mutated) phosphorylates CHEK2, which in turn phosphorylates p53 leading to cell cycle arrest and apoptosis (Iniesta et al. 2010). In addition, CHEK2 protein regulates BRCA1 in response to DNA damage (Oldenburg et al. 2007).

The CHEK2 1100delC protein truncating variant is situated in exon 10 of the functional gene on chromosome 22q. This variant is caused by the deletion of a single cytosine at position 1100 resulting in the introduction of a stop codon after amino acid 380, inducing a loss of activity of the CHEK2 kinase (Iniesta et al. 2010).

The CHEK2 1100delC variant was found in women suffering from breast cancer with familial Li-Fraumeni syndrome (Bell et al. 1999). In 2004, CHEK2-Breast Cancer Consortium did a collaborative analysis with 10 studies from 5 western countries, which involved 10 860 breast cancer cases and 9 065 controls (CHEK2 Breast Cancer Case–control Consortium CHEK2*1100delC and susceptibility to breast cancer 2004). The Consortium found the frequency of CHEK2 1100delC to be 1.9% and 0.7% in cases and controls respectively, and confirmed that this gene variant could potentially increase the risk of breast cancer. The CHEK2 1100delC variant has been reported to be a low-penetrance breast cancer susceptibility allele (Vahteristo et al. 2002; CHEK2 Breast Cancer Case–control Consortium CHEK2*1100delC and susceptibility to breast cancer 2004; Oldenburg et al. 2003; Kuusisto Kirsi et al. 2011). It results in an approximately two-fold risk of breast cancer in women and a ten-fold risk in men (van der Groep et al. 2011). It has been detected in 5% of breast cancer patients from non-BRCA1 and BRCA2 families (Vahteristo et al. 2002; Meijers-Heijboer et al. 2002). The frequency of CHEK2 1100delC allele varies among different populations. Indeed, high mutation rates are seen in Northern and Eastern European countries (Vahteristo et al. 2002; CHEK2 Breast Cancer Case–control Consortium CHEK2*1100delC and susceptibility to breast cancer 2004; Meijers-Heijboer et al. 2002) although its frequency is much lower in North America (Mateus Pereira et al. 2004; Offit et al. 2003), whereas the mutation does not seem to be a triggering factor to breast cancer in Poland (Kwiatkowska et al. 2006; Cybulski et al. 2004) and some multiple-case breast cancer families from Australia (Jekimovs et al. 2005). Likewise, the frequency of CHEK2 1100delC seems to be very low in Southern Europe, Italy (Caligo et al. 2004), Spain (Osorio et al. 2004; Bellosillo et al. 2005) and rare in Brazil (Zhang et al. 2008).

Moreover, another quantitative synthesis was done by Weischer et al. (Weischer et al. 2007). Combined with 16 studies, it showed that CHEK2 1100delC heterozygotes rate was 3- to 5-fold higher in the breast cancer group than the control group. However, this widely discussed variant of CHEK2 – which seemed clearly associated with the predominance of breast cancer in western countries – was rarely detected in Asian populations, such as the Chinese (Song et al. 2006), Koreans (Choi et al. 2008), Japanese (Bell et al. 2007), Singaporeans (Lee & Ang 2008), Malaysians (Thirthagiri et al. 2009) and South Indians (Rajkumar et al. 2003).

In Morocco, according to the Greater Casablanca Cancer Registry, breast cancer seems to be the first female cancer with a standardized incidence of 36.4 per 100,000 women for an average age of 49.5 years (Bouchbika et al. 2013). Although this incidence appears higher compared to the other Maghreb countries (Hamdi Cherif et al. 2010; Ben Abdallah et al. 2009; El Mistiri et al. 2010), it remains low compared to developed countries (Marrett et al. 2008; Botha et al. 2003; Jemal et al. 2011). In the greater area of Casablanca, 57 % of the cases were under 50 years of age (Bouchbika et al. 2013) and 7% of cases registered at the city of Rabat are younger than 35 years (Tazi et al. 2013).

In addition, Morocco is a country of northwestern Africa composed predominantly of Berber and Arab ethnic groups. The contribution of these ethnic groups to the genetic diversity of Moroccan population is evident. Therefore, it is crucial to improve our understanding, both on the subject of genetic susceptibilities and environmental risk factors. In the present study, we examined DNA from Moroccan patients who have been screened and found not to carry *BRCA1/2* mutations. The purpose of the study was to determine the frequency of CHEK2 1100delC mutation and the implication of CHEK2 as a breast cancer susceptibility gene in the Moroccan population.

## Materials and methods

### Subjects

DNA samples were collected from 134 patients. Among them, 113 were recruited from Mohammed VI Center for Cancer Treatment of Ibn Rochd University Hospital of Casablanca during 2009–2010 and 21 patients were kindly provided by the Oncology Unit of the Littoral Clinic during 2013.

**Figure 1:**
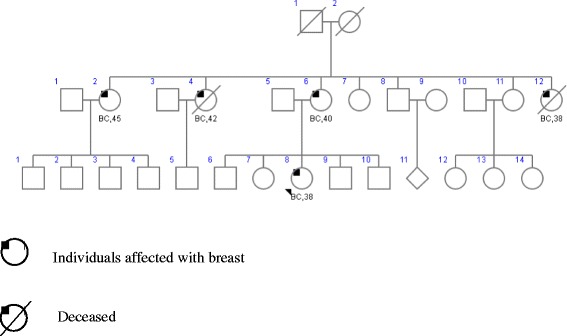
**Pedigree corresponding to one of the families included in the present study of CHEK2 1100delC mutation in hereditary breast cancer.** The index case is indicated with an arrow.

Data were collected from medical records and clinico-pathological reports. The group of sporadic cases (n = 75) presented a mean age of 36.9 years with an age range of 23–59 years. The group of familial breast and/or ovarian cases (n = 59) presented a mean age of 44.8 years with an age range of 25–67 years, and included women with specific family-history criteria (Figure 1):

- Three or more first or second degree relatives with breast cancer diagnosed in the same familial branch, at any age
- Two first degree relatives with breast cancer, with at least one early onset breast cancer case (≤40 years) or male breast cancer case or ovarian cancer case.

The control group included a total of 114 matched healthy women with no personal history of cancer diseases selected from DNA bank volunteers of the Genetics and Molecular Pathology Laboratory with a median age of 36.4 years and an age range of 20–77 years.

The study was approved by our institutional ethics committee and written informed consent for genetic testing was obtained from all subjects.

### DNA isolation and PCR amplification

Genomic DNA was extracted from peripheral blood leukocytes using the salting out method (Miller et al. 1988). Genomic DNA was dissolved in TE (10 mM Tris–HCl and 0.1 mM EDTA, pH 8.0), confirmed by agarose gel electrophoresis and then quantified using the Nanovue TM Plus spectrophotometer.

ASO-PCR analysis was performed to determine the 1100delC mutation of CHEK2 gene, as described previously (Rashid et al. 2005).The CHEK2 1100delC mutation genotyping was performed with forward primer CHEK2ex10f (5′-GCAAAATTAAATGTCCTAACTTGC-3′) in combination with previously described reverse primers CHEK2ex10r andCHEK2delCr primers (Cybulski et al. 2004).

CHEK2 gene was amplified in a final volume of 25 μl containing: 10× reaction buffer, 25 mM MgCl_2_, 5 mM dNTPs, 5 μM primers, 500 U HotStarTaq DNA polymerase and 150 ng genomic DNA. Touchdown PCR conditions were as follows: 15 min at 95°C, 10 cycles of 20 s at 94°C, 25 s at annealing temperature 68–55°C touchdown (1.4°C/cycle), and 30 s at 72°C followed by 36 cycles of 20 s at 94°C, 25 s at 55°C, and 35 s at 72°C, and then a final extension step of 10 min at 72°C.

The PCR product was separated by electrophoresis in a 1% agarose gel containing ethidium bromide and visualized under UV illumination. Wild type allele resulted in 537 bp fragment and variant allele resulted in 200 bp fragment.

## Results

A total of 134 Moroccan subjects were selected for our study. General characteristic of the subjects including distribution of tumor characteristic such as histological grade and location of cancer were obtained from patients’ medical records and listed in Table 1. In total, 134 breast cancer cases and 114 controls were successfully screened for the 1100delC variant of the CHEK2 gene using the ASO-PCR technique. None of the 134 Moroccan breast cancer patients carried the CHEK2 1100delC mutation. Moreover among the 134 cases, 40 patients had been screened for BRCA1 and BRCA2 mutations (Tazzite et al. 2012). Thus, in the 30 patients who were non-BRCA mutation carriers, the analysis of 1100delC mutation was also found negative.

**Table 1:** **Characteristics of individuals with breast cancer at time of diagnosis, screened for the CHEK2 1100delC mutation**

**Characteristics**	**Samples**
Cases/Controls	134 / 114
Age at diagnosis, mean ± SD (years)	41 ± 11
Range (years)	23– 67
Menopausal status	No. (%)
**Premenopausal**	76(56.71)
**Postmenopausal**	57(42.53)
**Missing**	1(0.74)
Estrogen receptor	
**Positive**	100 (74.62)
**Negative**	24(17.91)
**Missing**	10 (7.46)
Progesterone receptor	
**Positive**	98 (73.13)
**Negative**	30(22.38)
**Missing**	6 (4.47)
Estrogen/Progesterone receptor	
**ER** ^**+**^ **/PR** ^**+**^	66 (49.25)
**ER** ^**+**^ **/PR** ^**−**^	15 (11.19)
**ER** ^**−**^ **/PR** ^**+**^	10 (7.46)
**ER** ^**−**^ **/PR** ^**−**^	43 (32.08)
Tumor size	
**<2 cm**	20 (14.92)
**>2 cm**	56 (41.79)
**>5 cm**	31(23.13)
**Tumor of any size with extension**	27 (20.14)
Histological grade	
**1**	8 (5.97)
**2**	87 (64.92)
**3**	39 (29.10)
Lymph node status	
**Negative**	56(41.79)
**Positive**	78 (58.20)
Distant metastases	
**Negative**	106(79.10)
**Positive**	28 (20.89)

This finding suggests that this mutation is probably not present or is present at an extremely low frequency in Moroccan population.

## Discussion

In our present molecular study, we evaluated the involvement of the low-penetrance susceptibility 1100delC allelic variant in the CHEK2 gene in the Moroccan population. For this purpose, we performed a screening of this mutation in 134 Moroccan breast cancer patients and 114 healthy controls. Our results have shown that none of the 248 analyzed samples carried the CHEK2 1100delC mutation, suggesting that the frequency of this mutation is extremely low (or not present) in the Moroccan population.

On the other hand, our results are in line with some previous findings showing that there was no association between CHEK2 1100delC mutation and risk for Breast Cancer (Zhang et al. 2008; Caligo et al. 2004; Osorio et al. 2004; Bellosillo et al. 2005; Rajkumar et al. 2003; Sodha et al. 2007; Gonzalez-Hormazabal et al. 2008) (Table 2). Nonetheless, it should be noted that in Northern and Eastern European (Vahteristo et al. 2002; CHEK2 Breast Cancer Case–control Consortium CHEK2*1100delC and susceptibility to breast cancer 2004; Meijers-Heijboer et al. 2002) CHEK2 1100delC mutation is associated with an increased risk of Breast Cancer (on the basis of age at diagnosis and family history of breast cancer).

**Table 2:** **Frequency of CHEK2 1100delC mutation in breast cancer cases and controls by ethnicity**

			**Overall**	**Overall**	**Carriers n (frequency of carriers, %)**	**Carriers n (frequency of carriers, %)**	
**Study**	**Population**	**Year**	**Case**	**Control**	**Case**	**Control**	**Reference**
***América***							
1. Meijers-Heijboer and al	USA	2002	264	166	6 (2.3)	1(0.6)	(Meijers-Heijboer et al. 2002)
2. Offit KPH and al	USA	2003	67	569	0 (0)	2 (0.4)	(Offit et al. 2003)
3. Pereira and al	USA	2004	829	859	9 (1.1)	4(0.5)	(Mateus Pereira et al. 2004)
4. Friedrichsen and al	USA	2004	506	459	6 (1.2)	2 (0.4)	(Friedrichsen et al. 2004)
5. Bernstein and al	CANADA	2006	1199	496	18 (1.34)	1 (0.2)	(Bernstein et al. 2006)
6. Novak and al	CANADA	2008	149	141	3 (2.01)	1 (0.7)	(Novak et al. 2008)
7. Shiyu Zhang and al	CANADA	2008	560	6460	4 (0.7)	19 (0.3)	(Zhang et al. 2008)
8. Gonzalez-Hormazabal and al	CHILE	2008	196	1124	0 (0)	0 (0)	(Gonzalez-Hormazabal et al. 2008)
9. Shiyu Zhang and al	BRAZIL	2008	155	377	1 (0.7)	0 (0)	(Zhang et al. 2008)
10. Bell DW	LATINAS	2007	362	384	1	0 (0)	(Sodha et al. 2007)
***Europe***							
Sodha and al	UNITED KINGDOM	2002	68	300	3(4.4)	0(0)	(Sodha et al. 2002)
Check2 Consortium	UNITED KINGDOM	2004	2886	3749	35(1.2)	20 (0.53)	(CHEK2 Breast Cancer Case–control Consortium CHEK2*1100delC and susceptibility to breast cancer 2004)
Meijers-Heijboer and al	UNITED KINGDOM	2002	564	288	7(1.3)	1(0.35)	(Meijers-Heijboer et al. 2002)
Meijers-Heijboer and al	THE NETHERLANDS	2002	79	460	2(2.5)	6(1.2)	(Meijers-Heijboer et al. 2002)
Oldenburg and al	THE NETHERLANDS	2003	237	212	27(11.4)	6(2.8)	(Oldenburg et al. 2003)
Check2 Consortium	THE NETHERLANDS	2004	1706	184	65(3.8)	3(1.6)	(CHEK2 Breast Cancer Case–control Consortium CHEK2*1100delC and susceptibility to breast cancer 2004)
De Jong and al	THE NETHERLANDS	2005	962	367	28(2.9)	5(1.4)	(De Jong et al. 2005)
Offit KPH and al	ASHKENAZI JEWISH	2003	33	1096	1(3)	3(0.3)	(Offit et al. 2003)
Shiyu Zhang and al	JEWISH	2008a	320	180	4(1.3)	0(0)	(Zhang et al. 2008)
McInerney and al	IRELAND	2009	903	1016	5(0.5)	1(0.1)	(McInerney et al. 2009)
Martinez-Bouzas and al	BASQUE COUNTRY	2007	214	120	2(0.93)	0(0)	(Martınez-Bouzas et al. 2007)
Cybulski and al	POLAND	2004a	1017	4000	5(0.5)	10(0.25)	(Cybulski et al. 2004)
Cybulski and al	POLAND	2007b	4454	5496	20(0.4)	12(0.2)	(Cybulski et al. 2007)
Kleibl and al	CZECH REPUBLIC	2005	1046	730	4(0.38)	2(0.27)	(Kleibl et al. 2005)
Check2 Consortium	GERMANY	2004	985	401	11(1.1)	1(0.25)	(CHEK2 Breast Cancer Case–control Consortium CHEK2*1100delC and susceptibility to breast cancer 2004)
Dufault and al	GERMANY	2004	516	1315	8(1.6)	6(0.5)	(Dufault et al. 2004)
Rashid and al	GERMANY	2005	613	651	5(0.82)	6(0.92)	(Rashid et al. 2005)
Einarsdottir and al	SWEDEN	2006	1510	1334	20(0.7)	8(0.4)	(Einarsdottir et al. 2006)
Margollin and	SWEDEN	2007	450	760	10(2.2)	5(0.7)	(Margolin et al. 2007)
Weischer and al	DENMARK	2007	1088	4643	13(1.1)	22(0.5)	(Weischer et al. 2007)
Vahteristo and al	FINLAND	2002	1035	1885	21(2.1)	26 (1.4)	(Vahteristo et al. 2002)
Check2 Consortium	FINLAND	2004	464	447	13(2.9)	5(1.1)	(CHEK2 Breast Cancer Case–control Consortium CHEK2*1100delC and susceptibility to breast cancer 2004)
Kirsi and al	FINLAND	2011	82	380	3(0.037)	6(0.016)	(Kuusisto Kirsi et al. 2011)
Osorio and al	SPAIN	2004	456	400	0(0)	0(0)	(Osorio et al. 2004)
Caligo and al	ITALY	2004	939	334	1(0.1)	0(0)	(Caligo et al. 2004)
Baeyens and al	BELGIUM	2005	178	100	4(2.24)	0(0)	(Baeyens et al. 2005)
***Asia***							
Shiyu Zhang and al	FILIPINO	2008	342	7	0(0)	0(0)	(Zhang et al. 2008)
Chekmariova and al	RUSSIA	2006	815	448	22(5.2)	1(0.2)	(Chekmariova et al. 2006)
Bell DW	JAPANE	2007	428	378	0(0)	0(0)	(Sodha et al. 2007)
Rajkumar T and al	SOUTH INDIA	2003	22	1	0(0)	0(0)	(Rajkumar et al. 2003)
***Africa***							
Present study	MOROCCO	2013	134	114	0(0)	0(0)	Present study

A rational explanation for these discrepancies may be due to ethnic or geographic variations. As reported in several studies, it is evident that the contribution of CHEK2 1100delC mutation to the burden of cancer varies according to the ethnic group, and from country to country (Antoni et al. 2007; Gonzalez-Hormazabal et al. 2008; Martınez-Bouzas et al. 2007) (Table 2). For instance, CHEK2 1100delC mutation was frequently observed in some Western and Northern Europe (CHEK2 Breast Cancer Case–control Consortium CHEK2*1100delC and susceptibility to breast cancer 2004), but it was very rare in the Central Europe (Kleibl et al. 2005; Kwiatkowska et al. 2006), Southern Europe (Italy and Spain) (Caligo et al. 2004; Osorio et al. 2004) and Australia (Jekimovs et al. 2005). In Basque Country, Martinez-Bouzas et al. reported the 1100delC mutation in 0.93% of the cases with breast cancer, and in none of the control populations. Therefore, the authors raise the hypothesis of the existence of a 1100delC frequency gradient from the North-West to the South-East of Europe, caused by an ancestral common origin in the Northern Europe (Osorio et al. 2004; Gonzalez-Hormazabal et al. 2008; Martınez-Bouzas et al. 2007). Likewise, this variant is very low in North America (CHEK2 Breast Cancer Case–control Consortium CHEK2*1100delC and susceptibility to breast cancer 2004; Offit et al. 2003), and rare in Brazil (Zhang et al. 2008). The CHEK2 1100delc mutation is not present in Chilean families with familial breast (Gonzalez-Hormazabal et al. 2008), and it was rarely detected in Asian populations, such as the Chinese (Song et al. 2006; Chen et al. 2008), Koreans (Choi et al. 2008), Japanese (Sodha et al. 2007), Singaporeans (Lee & Ang 2008), Malysians (Thirthagiri et al. 2009), South Indians (Rajkumar et al. 2003), and Philippines (Zhang et al. 2008).

Antoniou et al. (2002) suggested that susceptibility to breast cancer in non-carriers of BRCA1 and BRCA2 mutations may be mainly due to a “polygenic” model, with a large number of susceptibility alleles, each conferring a small increase in risk. Further evaluation of these interactions will be required to identify and analyze other susceptibility genes. Moreover, Martınez-Bouzas et al. (2007) detected a 0,93% prevalence among 214 Basque Country non BRCA1/2 patients and Gutiérrez-Enríquez et al. (2008)) reported 0,3% frequency in 331 non BRCA1/2 families from Basque Country and Catalonia. On the other hand, Zhang et al. (2008) found no CHEK2 mutation in 307 White women with breast cancer and a BRCA mutation. It is unlikely that women with a BRCA mutation will be found to harbor a CHEK2 mutation.

However, it is possible that other *CHEK2* variants will confer susceptibility to breast cancer in other countries. Therefore, many studies screened the full coding sequence of CHEK2. For example, a splice-site mutation in *CHEK2* IVS2 + G > A has been found to be associated with breast cancer susceptibility in Poland (Cybulski et al. 2007), and the S428F allele of the CHEK2 gene increases breast cancer risk in Ashkenazi Jewish women (Shaag et al. 2005). Likewise, several previous studies have suggested that CHEK2 I157T variant may contribute to inherited breast cancer predisposition (Cybulski et al. 2007; Kilpivaara et al. 2004; Bogdanova et al. 2005).

A limitation of our study was the reliance on family members’ reports of cancer in their relatives. Secondly, the sample size was limited and it is important to confirm our findings in a larger study. Thirdly, this study only focused on single gene without taking into consideration SNP-SNP and gene–gene interactions, or the possibility of linkage disequilibrium between polymorphisms, which may affect individual susceptibility to breast cancer.

## Conclusion

Since its discovery as a BC susceptibility allele, the occurrence of CHEK2 1100delC mutation depends on the geographical area and/or ethnical characteristics of populations (CHEK2 Breast Cancer Case–control Consortium CHEK2*1100delC and susceptibility to breast cancer 2004). Thus, the absence or rarity of the CHEK2*1100delC heterozygosity among patients with breast cancer in our population underlines the importance of considering ethnic background before offering a genetic test. Further studies need to expand and elaborate on the putative contribution of this variant in the Moroccan population.
